# Effect of setup error in the single‐isocenter technique on stereotactic radiosurgery for multiple brain metastases

**DOI:** 10.1002/acm2.13081

**Published:** 2020-10-29

**Authors:** Hisashi Nakano, Satoshi Tanabe, Satoru Utsunomiya, Takumi Yamada, Ryuta Sasamoto, Toshimichi Nakano, Hirotake Saito, Takeshi Takizawa, Hironori Sakai, Atsushi Ohta, Eisuke Abe, Motoki Kaidu, Hidefumi Aoyama

**Affiliations:** ^1^ Department of Radiation Oncology Niigata University Medical and Dental Hospital Chuo‐ku Niigata Japan; ^2^ Department of Radiological Technology Niigata University Graduate School of Health Sciences Chuo‐ku Niigata Japan; ^3^ Section of Radiology Department of Clinical Support Niigata University Medical and Dental Hospital Chuo‐ku Niigata Japan; ^4^ Department of Radiology and Radiation Oncology Niigata University Graduate School of Medical and Dental Sciences Chuo‐ku Niigata Japan; ^5^ Department of Radiation Oncology Niigata Neurosurgical Hospital Nishi‐ku Niigata Japan; ^6^ Department of Radiology Nagaoka Chuo General Hospital Nagaoka Japan; ^7^ Department of Radiation Oncology Faculty of Medicine Hokkaido University Kita‐ku Sapporo Japan

**Keywords:** multiple brain metastases, setup error, single‐isocenter technique, stereotactic radiosurgery, volumetric modulated arc therapy

## Abstract

In conventional stereotactic radiosurgery (SRS), treatment of multiple brain metastases using multiple isocenters is time‐consuming resulting in long dose delivery times for patients. A single‐isocenter technique has been developed which enables the simultaneous irradiation of multiple targets at one isocenter. This technique requires accurate positioning of the patient to ensure optimal dose coverage. We evaluated the effect of six degrees of freedom (6DoF) setup errors in patient setups on SRS dose distributions for multiple brain metastases using a single‐isocenter technique. We used simulated spherical gross tumor volumes (GTVs) with diameters ranging from 1.0 to 3.0 cm. The distance from the isocenter to the target's center was varied from 0 to 15 cm. We created dose distributions so that each target was entirely covered by 100% of the prescribed dose. The target's position vectors were rotated from 0°–2.0° and translated from 0–1.0 mm with respect to the three axes in space. The reduction in dose coverage for the targets for each setup error was calculated and compared with zero setup error. The calculated margins for the GTV necessary to satisfy the tolerance values for loss of GTV coverage of 3% to 10% were defined as coverage‐based margins. In addition, the maximum isocenter to target distance for different 6DoF setup errors was calculated to satisfy the tolerance values. The dose coverage reduction and coverage‐based margins increased as the target diameter decreased, and the distance and 6DoF setup error increased. An increase in setup error when a single‐isocenter technique is used may increase the risk of missing the tumor; this risk increases with increasing distance from the isocenter and decreasing tumor size.

## 1 INTRODUCTION

Volumetric‐modulated arc therapy (VMAT) is a modification of intensity‐modulated radiation therapy. It produces a highly accurate three‐dimensional dose distribution with single or multi‐arc irradiation using a 360° gantry rotation.^1^, ^2^, ^3^ VMAT generates dose distributions using dynamic multi‐leaf collimators and variable dose rates and gantry speeds.^4^, ^5^ By optimizing the dose distributions, the VMAT irradiation technique provides both a highly controlled dose to the target(s) and a reduction of the dose to normal tissue(s).^6^ Cranial stereotactic radiosurgery (SRS) administered with a linear accelerator has been used with multiple isocenters to treat multiple brain metastases.^7^, ^8^ In conventional SRS, when using multiple isocenters for multiple brain metastases, one isocenter is set for one target, resulting in a long dose delivery time for patients^9^, ^10^ which is a disadvantage of such treatment. A single‐isocenter VMAT (SIVMAT) technique was introduced for multiple brain metastases.^10^, ^11^, ^12^ This technique enables the simultaneous irradiation of multiple targets at one isocenter, thus making it possible to significantly shorten the dose delivery time and overcome the disadvantage of conventional SRS. In addition, SIVMAT can deliver equivalent dose conformity for each target^13^, ^14^ and reduce the dose for normal tissues such as healthy brain tissue, utilizing the advantages of VMAT without the disadvantages of multiple‐isocenter SRS.^15^, ^16^

However, the patient setup accuracy for a SIVMAT treatment has a greater impact on the dose distribution than multiple‐isocenter VMAT because the planning isocenter is not necessarily located at the center of the targets in many instances.^7^, ^8^, ^9^, ^10^, ^11^ In SIVMAT, it is believed that the effect of any rotational error in a patient setup is dependent on the relationship between the diameter of the target and the distance from the isocenter to the target. In multiple‐isocenter irradiation the distance from the isocenter to the target does not need to be considered.^17^, ^18^ In addition, the dose coverage is affected by any translational error in the setup.^19^, ^20^ A six degrees of freedom (6DoF) setup error is determined by adding the translational error to the rotational error. It is important to assess the effect of this error on the dose coverage for gross tumor volumes (GTVs) by varying the distance from the isocenter to the target, and varying the target size; however, there have been few studies that made this evaluation for SIVMAT, and none that the margin is calculated to satisfy the tolerance values of dose coverage reduction for GTV. In SIVMAT, the dose coverage is affected by the 6DoF setup error, the distance from the isocenter to the targets, and the target diameter. Therefore, larger planning target volume (PTV) margins considering the 6DoF setup error were thought to be necessary in SIVMAT compared to those needed for multiple‐isocenter irradiation.

In SRS, a 0.1‐cm PTV margin is often used in clinical settings to concentrate the radiation on the GTV and to minimize the doses to surrounding normal tissue.^21^, ^22^ The effect of the rotational error becomes more significant as the distance between the target and the isocenter increases. It has been speculated that the clinical PTV margin of 0.1 cm cannot be secured for dose coverage in SIVMAT. It is therefore important to determine the maximum distance between the isocenter and target that allows the clinical PTV margin to secure the dose coverage for the GTV with varying 6DoF setup error. In this study, we calculated the PTV margin for GTVs that is required to satisfy the various tolerance values when a 6DoF setup error occurs.

Furthermore, we calculated the maximum distance between the isocenter and the target with which a clinical PTV margin secures the tolerance values of dose coverage reduction in SIVMAT by using the derivation of coverage‐based margin.

## 2 MATERIALS AND METHODS

### 2.A Phantom design

**Fig. 1:**
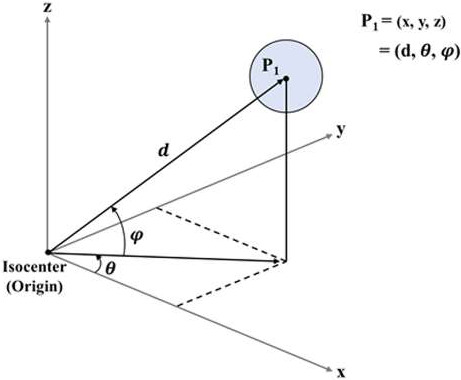
Locations of the isocenter (the origin of the coordinate axes) and the target in Cartesian coordinates *P_1_* [x, y, z].

The diameters of the simulated GTVs were set as follows: 1.0 cm (GTV 1), 1.5 cm (GTV 2), 2.0 cm (GTV 3), and 3.0 cm (GTV 4) with MATLAB ver. 2019a software (MathWorks, Natick, MA, USA). The coordinates (unit: cm) of the GTVs were set such that the distance between the center of the GTV and the isocenter varied from 0 to 15 cm.^19^ The isocenter was set as the origin of the coordinate axes. We created the dose distribution vectors so that each target was entirely covered by 100% of the prescribed dose. As shown in Fig. 1, the axis of rotation was defined by the origin of the rotation and point *P_1_*. The spherical coordinates are the Cartesian coordinates, that is, x, y, and z. Equation (1) shows the conversion of polar coordinates to the Cartesian coordinate system.

(1) $$\begin{array}{c} x=d\cos\varphi \cos\theta \\ y=d\cos\varphi \sin\theta \\ z=d\sin\varphi \end{array}$$

### 2.B Dose coverage reduction with rotational error

The point *P*_1_ (x, y, z) is rotated around the x‐axis by an angle α, around the y‐axis by an angle β, and around the z‐axis by an angle γ to obtain *P_2_* (x_rot_, y_rot_, z_rot_) in Eq. (2). The rotational angles of α, β, and γ were the same, and this value was defined as δ_rot_ (δ_rot_ = α, β, γ) in this study.

(2) $$\left(\begin{array}{c} x_{\mathrm{rot}}\\ y_{\mathrm{rot}}\\ z_{\mathrm{rot}} \end{array}\right)=\left(\begin{array}{ccc} \cos\gamma & \sin\gamma & 0\\ ‐\sin\gamma & \cos\gamma & 0\\ 0 & 0 & 1 \end{array}\right)\left(\begin{array}{ccc} \cos\beta & 0 & ‐\sin\beta \\ 0 & 1 & 0\\ \sin\beta & 0 & \cos\beta \end{array}\right)\left(\begin{array}{ccc} 1 & 0 & 0\\ 0 & \cos\alpha & \sin\alpha \\ 0 & ‐\sin\alpha & \cos\alpha \end{array}\right)\left(\begin{array}{c} x\\ y\\ z \end{array}\right)$$

The vectors of the target position coordinates with the isocenter as a start point were simultaneously rotated clockwise around the x‐, y‐, and z‐axes with δ ranging from 0° to 2.0° in 0.5° increments.

### 2.C Dose coverage reduction with 6DoF setup error

The 6DoF setup error was calculated by adding a translational error to *P_2_* (x_rot_, y_rot_, z_rot_). *P_3_* (x_setup_, y_setup_, z_setup_) was therefore calculated as the translational error δ_trans_ in the positive direction of the x‐, y‐, and z‐axes added to *P_2_* (x_rot_, y_rot_, z_rot_) [Eq. (3)]. The translational error δ_trans_ component values were 0.3, 0.5, and 1.0 mm.

(3) $$\left(\begin{array}{c} x_{\mathrm{setup}}\\ y_{\mathrm{setup}}\\ z_{\mathrm{setup}} \end{array}\right)=\left(\begin{array}{c} x_{\mathrm{rot}}+\delta_{\mathrm{xtrans}}\\ y_{\mathrm{rot}}+\delta_{\mathrm{ytrans}}\\ z_{\mathrm{rot}}+\delta_{\mathrm{ztrans}} \end{array}\right)$$

**Fig. 2:**
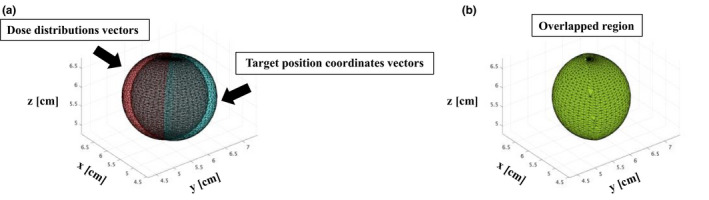
The overlapped region of rotated position coordinate vectors and dose distribution vectors: (a) as extracted, and (b) the calculated volume. The dose distributions, target position coordinates, and overlapped region are shown by a polyhedron consisting of three‐dimensional points meeting the following conditions: distance from the isocenter = 10 cm, diameter = 2.0 cm, rotational error = 2.0°, and translational error = 1.0 mm.

We extracted the overlapped region of the vectors of the rotated only and rotated and translated position coordinates based on the isocenter and the dose distribution vectors, and then calculated the volume of the overlapped area (Fig. 2). A polyhedron consisting of three‐dimensional points was used for the calculation of the overlapped volume (MATLAB). We calculated the reduction in dose coverage for the targets at each rotational error and 6DoF setup error. The results were then compared to those with 0° rotational error and 0 mm translational error for different values of the distance to the target center from the isocenter and the GTV diameter. In this study, the tolerance value of the dose coverage reduction for each GTV diameter was defined as a 3%, 5%, or 10% reduction in the prescription dose.

### 2.D Evaluation of coverage‐based margin for different GTV diameters

We defined the conditions under which the reduction in dose coverage for a GTV as being acceptable if the reduction was less than each tolerance value with a 0 cm margin. We extracted the conditions exceeding each tolerance value for the reduction of dose coverage. The diameter of the target corresponding to each tolerance value reduction (3%, 5%, and 10%) was introduced with the distance and 6DoF setup error when the reduction rate of dose coverage was greater than each tolerance value. In this study, the size of the margin required for the target to meet the requirement of each tolerance value reduction was defined as the coverage‐based margin. The coverage‐based margin was calculated for each GTV diameter, different distances from the GTV center to the isocenter, and different 6DoF setup errors based on the tolerance values.

### 2.E Maximum distance of clinical PTV margin

We defined the “clinical PTV margin” as the 0.1‐cm PTV margin added to the GTV diameter. The clinical PTV margin was needed to satisfy the 3%, 5%, and 10% tolerance values of dose coverage reduction as 6DoF setup error was introduced. The limiting scenarios where the dose coverage reduction tolerances could not be met, were studied as a function of distance from isocenter, GTV diameter, and 6DoF setup error. Specifically, the maximum distance at which the clinical PTV margin secures the tolerance values was calculated for each GTV diameter with set 6DoF setup errors, using the derivation of coverage‐based margins.

## 3 RESULTS

### 3.A Relationship between isocenter distance and dose coverage reduction with various rotational errors

**Fig. 3:**
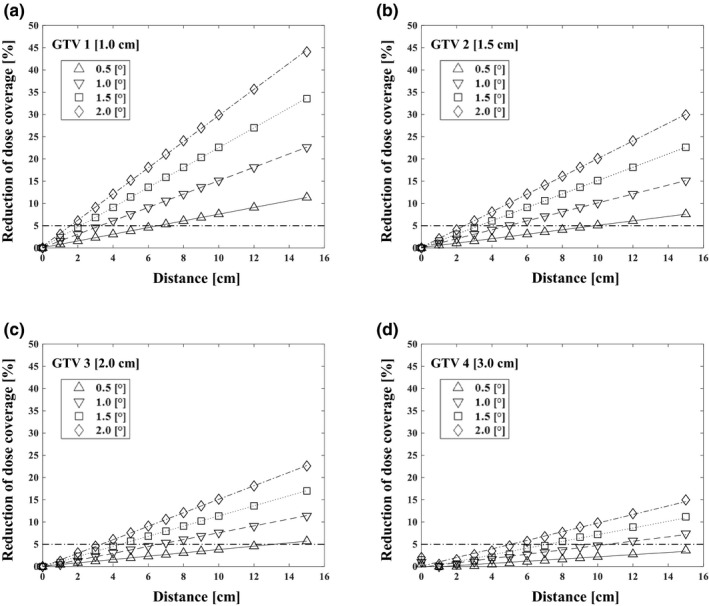
Relationship between distance from the isocenter and dose coverage reduction for different gross tumor volume (GTV) diameters and rotational errors with reference to a 5% tolerance value. The effects on the targets with diameters of 1.0 cm (GTV 1) (a), 1.5 cm (GTV 2) (b), 2.0 cm (GTV 3) (c), and 3.0 cm (GTV 4) (d) are shown.

We first evaluated the effect of only the rotational error on the dose coverage. We observed that the dose coverage of each target decreased as the rotational error increased, and a greater reduction in dose coverage occurred when the distance from the isocenter was increased and the target was smaller (Fig. 3). The reduction of dose coverages for GTV 1 were 3.8% and 11.4% when (d, δ_rot_) = (5 cm, 0.5°) and (15 cm, 0.5°). Those for GTV 2 were 2.6% and 7.6%, for GTV 3 were 1.9% and 5.7% and for GTV 4 were 1.3% and 3.8% (Table 1).

**Table 1:** The reduction of dose coverage [%] as a function of the diameter of the target, the distance from the isocenter to the target, and the rotational angle.

GTV dia.	Distance from isocenter to target	Rotational angle			
		0.5°	1.0°	1.5°	2.0°
1.0 cm (GTV 1)	1.0 cm	0.8	1.6	2.3	3.1
	3.0 cm	2.3	4.6	6.8	9.1
	5.0 cm	3.8	7.6	11.5	15.2
	10.0 cm	7.6	15.1	22.6	29.9
	15.0 cm	11.4	22.6	33.5	44.1
1.5 cm (GTV 2)	1.0 cm	0.6	1.1	1.6	2.1
	3.0 cm	1.6	3.1	4.6	6.1
	5.0 cm	2.6	5.1	7.6	10.1
	10.0 cm	5.1	10.1	15.1	20.1
	15.0 cm	7.6	15.1	22.6	29.9
2.0 cm (GTV 3)	1.0 cm	0.4	0.8	1.2	1.6
	3.0 cm	1.2	2.3	3.4	4.6
	5.0 cm	1.9	3.8	5.7	7.6
	10.0 cm	3.8	7.6	11.4	15.1
	15.0 cm	5.7	11.4	17.0	22.6
3.0 cm (GTV 4)	1.0 cm	0.4	0.6	0.9	1.1
	3.0 cm	0.8	1.6	2.3	3.1
	5.0 cm	1.3	2.6	3.8	5.1
	10.0 cm	2.6	5.1	7.6	10.1
	15.0 cm	3.8	7.6	11.4	15.1

### 3.B Relationship between isocenter distance and dose coverage reduction with different 6DoF setup errors

**Fig. 4:**
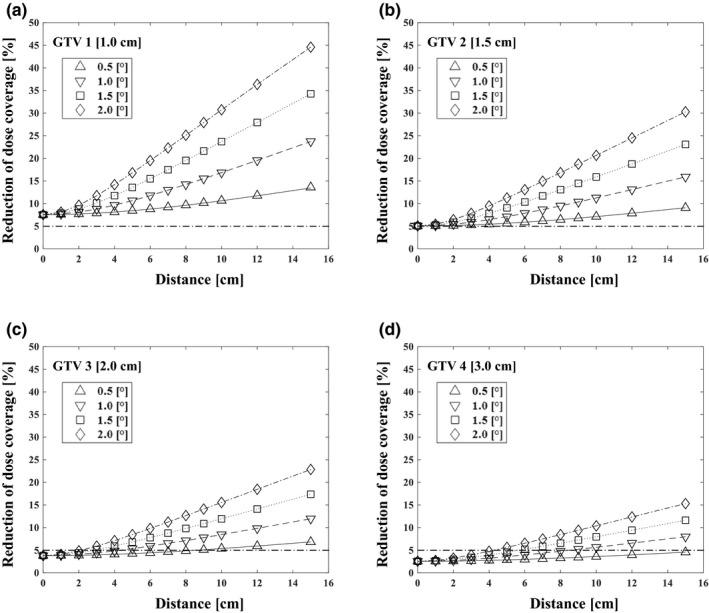
Relationship between distance from the isocenter and dose coverage reduction for different gross tumor volume diameters and rotational errors with translational error of 0.5 mm, with reference to a 5% tolerance value. The effects on the targets with diameters of 1.0 cm (GTV 1) (a), 1.5 cm (GTV 2) (b), 2.0 cm (GTV 3) (c), and 3.0 cm (GTV 4) (d) are shown.

We calculated the effect of 6DoF setup errors on the target dose coverage. After introducing 6DoF setup errors (various rotational error values and 0.5 mm translational error), the reduction in dose coverage of each target worsened with distance to isocenter and the decrease in target size (Fig. 4). Additional impact of additional translational errors are included in Table 2. Compared to the case of only the rotational error, the reduction in dose coverage was larger when considering the 6DoF setup error, and the tendency was more pronounced when the target diameter was smaller and the distance and 6DoF setup error were larger.

**Table 2:** The reduction of dose coverage [%] as a function of the diameter of the target, the distance from the isocenter to the target, and 6‐axis setup error.

GTV dia.	Distance from isocenter to target	Translational error											
		0.3 mm				0.5 mm				1.0 mm			
		Rotational error											
		0.5°	1.0°	1.5°	2.0°	0.5°	1.0°	1.5°	2.0°	0.5°	1.0°	1.5°	2.0°
1.0 cm (GTV 1)	1.0 cm	4.6	4.8	5.1	5.4	7.6	7.7	7.9	8.1	15.0	15.1	15.1	15.3
	3.0 cm	5.1	6.4	8.2	10.1	7.9	8.8	10.1	11.8	15.2	15.6	16.4	17.4
	5.0 cm	5.9	8.8	12.2	15.7	8.4	10.7	13.6	16.8	15.5	16.7	18.7	21.1
	10.0 cm	8.8	15.8	23.0	30.2	10.7	16.8	23.7	30.7	16.8	21.1	26.9	33.1
	15.0 cm	12.2	23.0	33.8	44.3	13.6	23.7	34.3	44.6	18.7	26.9	36.4	46.1
1.5 cm (GTV 2)	1.0 cm	3.1	3.2	3.4	3.6	5.1	5.2	5.3	5.4	10.0	10.1	10.1	10.2
	3.0 cm	3.4	4.3	5.5	6.8	5.3	5.9	6.8	7.9	10.1	10.4	11.0	11.6
	5.0 cm	4.0	5.9	8.1	10.5	5.7	7.1	9.1	11.2	10.3	11.2	12.5	14.1
	10.0 cm	5.9	10.5	15.4	20.3	7.1	11.3	15.9	20.7	11.2	14.2	18.0	22.3
	15.0 cm	8.2	15.4	22.8	30.0	9.1	15.9	23.1	30.3	12.5	18.0	24.6	31.4
2.0 cm (GTV 3)	1.0 cm	2.3	2.4	2.6	2.7	3.8	3.9	4.0	4.1	7.5	7.6	7.6	7.7
	3.0 cm	2.6	3.2	4.1	5.1	4.0	4.4	5.1	5.9	7.6	7.9	8.2	8.7
	5.0 cm	3.0	4.4	6.1	7.9	4.3	5.4	6.8	8.4	7.8	8.4	9.4	10.6
	10.0 cm	4.4	7.9	11.6	15.3	5.4	8.5	12.0	15.6	8.4	10.6	13.6	16.8
	15.0 cm	6.1	11.6	17.1	22.7	6.8	12.0	17.4	22.9	9.4	13.6	18.5	23.7
3.0 cm (GTV 4)	1.0 cm	1.6	1.6	1.7	1.9	2.6	2.6	2.7	2.7	5.0	5.1	5.1	5.1
	3.0 cm	1.7	2.2	2.8	3.4	2.7	3.0	3.4	4.0	5.1	5.3	5.5	5.9
	5.0 cm	2.0	3.0	4.1	5.3	2.9	3.6	4.6	5.6	5.2	5.6	6.3	7.1
	10.0 cm	3.0	5.3	7.7	10.2	3.6	5.7	8.0	10.4	5.6	7.1	9.1	11.2
	15.0 cm	4.1	7.7	11.5	15.2	4.6	8.0	11.6	15.3	6.3	9.1	12.4	15.9

The reduction of dose coverages for GTV 1 were 8.4% and 13.6% when (d, δ_rot_, δ_trans_) = (5 cm, 0.5°, 0.5 mm) and (15 cm, 0.5°, 0.5 mm). Those for GTV 2 were 5.7% and 9.1%, for GTV 3 were 4.3% and 6.8%. and for GTV 4 were 2.9% 4.6% (Table 2). For GTV 1 and GTV 2, the 3% and 5% tolerance values of dose coverage reduction were never satisfied, even when the distance from the isocenter was zero.

### 3.C Coverage‐based margin for different tolerance values

**Fig. 5:**
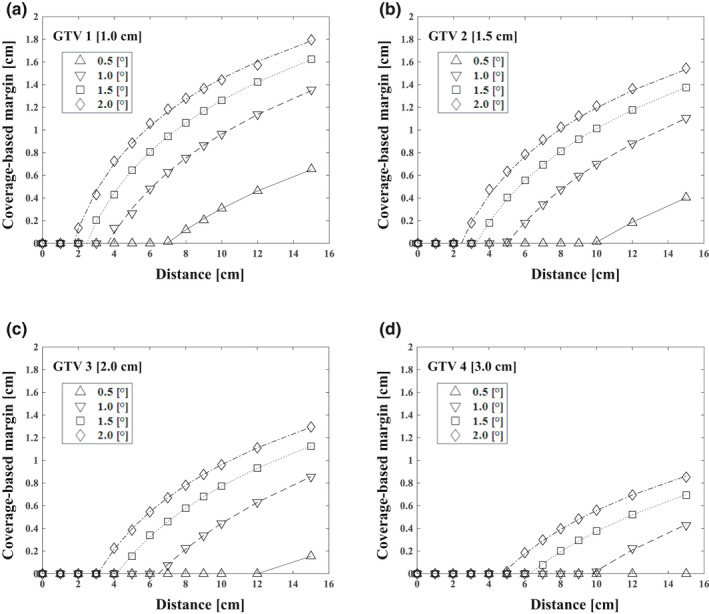
Relationship between the distance from the isocenter and the coverage‐based margin for various gross tumor volume diameters and rotational errors with 0.5 mm translational error to secure 5% tolerance value of dose coverage reduction. The effects on the targets with diameters of 1.0 cm (GTV 1) (a), 1.5 cm (GTV 2) (b), 2.0 cm (GTV 3) (c), and 3.0 cm (GTV 4) (d) are shown.

The coverage‐based margin was calculated for each GTV diameter, distance from the target to the isocenter, and 6DoF setup error based on the tolerance values. The coverage‐based margin that satisfied the 5% tolerance values increased as the 6DoF setup error increased, and a greater reduction in dose coverage was obtained when the distance was longer and the diameter of the target was smaller (Fig. 5). The coverage‐based margin for GTV 1 and GTV 2 on 5% tolerance values were 0.3, 0.7, 0.1, and 0.5 mm when (d, δ_rot_, δ_trans_) = (5 cm, 0.5°, 0.5 mm) and (15 cm, 0.5°, 0.5 mm), respectively.

The relationship between the coverage‐based margin for each tolerance value and 6DoF setup error is summarized in Tables 3, 4, and 5 which show the coverage‐based margins for 3%, 5%, and 10% tolerance values, respectively. The larger the tolerance value, the smaller the required coverage‐based margin. A large margin was needed to satisfy the 3% and 5% tolerance values. When the tolerance was 10%, the coverage loss met the tolerance value under most conditions. Under many of the conditions, that did require a margin to meet the 10% threshold, the margin was within what is clinically acceptable in SRS.

**Table 3:** The coverage‐based margins [cm] that satisfy each tolerance value with various target diameters, distances from the isocenter to the target, and setup errors for 3% as the tolerance value of dose coverage reduction for the GTV.

GTV dia.	Distance	Translational error															
		0 mm				0.3 mm				0.5 mm				1.0 mm			
		Rotational error															
		0.5°	1.0°	1.5°	2.0°	0.5°	1.0°	1.5°	2.0°	0.5°	1.0°	1.5°	2.0°	0.5°	1.0°	1.5°	2.0°
1.0 cm (GTV 1)	1.0 cm	0	0	0	0	0	0	0	0	0.8	0.8	0.8	0.8	1.4	1.4	1.4	1.4
	3.0 cm	0	0	0.2	0.3	0.3	0.3	0.3	0.3	0.8	0.9	0.9	0.9	1.4	1.4	1.4	1.4
	5.0 cm	0	0.5	0.5	0.7	0.3	0.4	0.6	0.8	0.9	1.0	1.3	1.4	1.4	1.5	1.5	1.5
	10.0 cm	0.4	1.2	1.3	1.5	0.9	1.4	1.6	1.7	1.1	1.4	1.6	1.7	1.5	1.6	1.7	1.7
	15.0 cm	0.6	1.2	1.5	1.6	1.1	1.5	1.7	1.8	1.3	1.6	1.7	1.8	1.5	1.6	1.7	1.8
1.5 cm (GTV 2)	1.0 cm	0	0	0	0	0	0	0	0	0.6	0.6	0.6	0.6	1.2	1.2	1.2	1.2
	3.0 cm	0	0	0.2	0.4	0.1	0.2	0.4	0.6	0.6	0.7	0.7	0.8	1.2	1.2	1.2	1.2
	5.0 cm	0.1	0.3	0.5	0.7	0.2	0.6	0.9	1.1	0.7	0.8	1.0	1.1	1.2	1.2	1.2	1.3
	10.0 cm	0.4	0.6	1.0	1.2	0.7	0.7	1.1	1.3	0.9	1.2	1.1	1.3	1.2	1.3	1.4	1.5
	15.0 cm	0.6	1.0	1.1	1.3	0.9	1.3	1.4	1.5	1.0	1.3	1.4	1.5	1.2	1.4	1.5	1.5
2.0 cm (GTV 3)	1.0 cm	0	0	0	0	0	0	0	0	0.3	0.3	0.3	0.3	0.9	0.9	0.9	0.9
	3.0 cm	0	0	0	0.1	0	0	0.1	0.3	0.4	0.4	0.5	0.6	0.9	0.9	0.9	1.0
	5.0 cm	0	0.1	0.3	0.7	0	0.3	0.6	0.8	0.4	0.6	0.8	0.9	0.9	1.0	1.0	1.0
	10.0 cm	0.2	0.5	0.9	1.0	0.4	0.9	1.1	1.2	0.6	0.9	1.1	1.2	1.0	1.1	1.2	1.2
	15.0 cm	0.3	0.9	1.0	1.2	0.6	1.0	1.2	1.3	0.8	1.1	1.2	1.3	1.0	1.1	1.2	1.3
3.0 cm (GTV 4)	1.0 cm	0	0	0	0	0	0	0	0	0	0	0	0	0.4	0.4	0.4	0.4
	3.0 cm	0	0	0	0	0	0	0	0	0	0	0	0.1	0.4	0.4	0.4	0.5
	5.0 cm	0	0	0	0.1	0	0	0.1	0.3	0	0.1	0.3	0.4	0.4	0.4	0.5	0.5
	10.0 cm	0	0.2	0.4	0.5	0	0.4	0.6	0.7	0.1	0.4	0.6	0.7	0.5	0.6	0.7	0.7
	15.0 cm	0	0.3	0.5	0.7	0.1	0.5	0.7	0.8	0.3	0.6	0.7	0.8	0.5	0.6	0.7	0.8

**Table 4:** The coverage‐based margins [cm] that satisfy each tolerance value with various target diameters, distances from the isocenter to the target, and setup errors for 5% as the tolerance value of dose coverage reduction for the GTV.

GTV dia.	Distance	Translational error															
		0 mm				0.3 mm				0.5 mm				1.0 mm			
		Rotational error															
		0.5°	1.0°	1.5°	2.0°	0.5°	1.0°	1.5°	2.0°	0.5°	1.0°	1.5°	2.0°	0.5°	1.0°	1.5°	2.0°
1.0 cm (GTV 1)	1.0 cm	0	0	0	0	0	0	0	0	0.3	0.3	0.3	0.3	1.0	1.0	1.0	1.0
	3.0 cm	0	0	0.2	0.3	0	0.1	0.2	0.3	0.3	0.3	0.4	0.5	1.0	1.0	1.1	1.1
	5.0 cm	0	0.3	0.5	0.4	0.1	0.3	0.5	0.8	0.3	0.5	0.7	1.0	1.0	1.1	1.2	1.2
	10.0 cm	0.3	0.9	1.2	1.4	0.3	1.0	1.3	1.5	0.5	1.0	1.3	1.5	1.1	1.3	1.4	1.5
	15.0 cm	0.5	1.1	1.4	1.5	0.5	1.2	1.5	1.6	0.7	1.2	1.5	1.6	1.2	1.4	1.5	1.6
1.5 cm (GTV 2)	1.0 cm	0	0	0	0	0	0	0	0	0	0	0	0	0.7	0.7	0.7	0.7
	3.0 cm	0	0	0	0	0	0	0	0	0	0.1	0.2	0.2	0.7	0.8	0.8	0.8
	5.0 cm	0	0	0.2	0.5	0	0	0.3	0.6	0.1	0.2	0.5	0.7	0.8	0.8	0.9	1.0
	10.0 cm	0	0.6	1.0	1.2	0.1	0.7	1.0	1.2	0.3	0.8	1.1	1.2	0.8	1.0	1.2	1.3
	15.0 cm	0.3	1.0	1.1	1.3	0.3	1.0	1.2	1.4	0.5	1.0	1.2	1.4	0.19	1.1	1.3	1.4
2.0 cm (GTV 3)	1.0 cm	0	0	0	0	0	0	0	0	0	0	0	0	0.5	0.5	0.5	0.5
	3.0 cm	0	0	0	0	0	0	0	0	0	0	0	0	0.5	0.5	0.6	0.6
	5.0 cm	0	0	0	0.5	0	0	0	0.3	0	0	0.2	0.5	0.5	0.6	0.7	0.7
	10.0 cm	0	0.4	0.7	0.9	0	0.5	0.8	1.0	0	0.5	0.8	1.0	0.6	0.8	0.9	1.0
	15.0 cm	0	0.7	0.9	1.0	0	0.7	1.0	1.1	1.2	0.2	0.7	1.0	0.7	0.9	1.0	1.1
3.0 cm (GTV 4)	1.0 cm	0	0	0	0	0	0	0	0	0	0	0	0	0	0	0	0
	3.0 cm	0	0	0	0	0	0	0	0	0	0	0	0	0	0	0.1	0.1
	5.0 cm	0	0	0	0	0	0	0	0	0	0	0	0	0	0.1	0.2	0.2
	10.0 cm	0	0	0.2	0.4	0	0	0.3	0.5	00	0	0.3	0.5	0.1	0.3	0.4	0.5
	15.0 cm	0	0.1	0.4	0.6	0	0.2	0.5	0.6		0.2	0.5	0.6	0.2	0.5	0.5	0.6

**Table 5:** The coverage‐based margins [cm] that satisfy each tolerance value with various target diameters, distances from the isocenter to the target, and setup errors for 10% as the tolerance value of dose coverage reduction for the GTV.

GTV dia.	Distance	Translational error															
		0 mm				0.3 mm				0.5 mm				1.0 mm			
		Rotational error															
		0.5°	1.0°	1.5°	2.0°	0.5°	1.0°	1.5°	2.0°	0.5°	1.0°	1.5°	2.0°	0.5°	1.0°	1.5°	2.0°
1.0 cm (GTV 1)	1.0 cm	0	0	0	0	0	0	0	0	0	0	0	0	0.3	0.3	0.3	0.3
	3.0 cm	0	0	0	0	0	0	0	0	0	0	0	0	0.3	0.3	0.3	0.3
	5.0 cm	0	0	0	0	0	0	0	0	0	0	0.1	0.2	0.3	0.3	0.4	0.5
	10.0 cm	0	0	0.2	0.4	0	0.2	0.6	0.9	0	0.3	0.7	1.0	0.3	0.5	0.8	1.0
	15.0 cm	0	0.3	0.6	1.0	0	0.5	0.9	1.2	0.1	0.6	1.0	1.3	0.4	0.7	1.1	1.4
1.5 cm (GTV 2)	1.0 cm	0	0	0	0	0	0	0	0	0	0	0	0	0	0	0	0
	3.0 cm	0	0	0	0	0	0	0	0	0	0	0	0	0	0	0	0.1
	5.0 cm	0	0	0	0	0	0	0	0	0	0	0	0	0	0.1	0.2	0.2
	10.0 cm	0	0	0	0.2	0	0	0.3	0.7	0	0	0.4	0.7	0.1	0.3	0.6	0.8
	15.0 cm	0	0	0.3	0.4	0	0.2	0.7	0.9	0	0.3	0.8	0.9	0.1	0.5	0.8	1.0
2.0 cm (GTV 3)	1.0 cm	0	0	0	0	0	0	0	0	0	0	0	0	0	0	0	0
	3.0 cm	0	0	0	0	0	0	0	0	0	0	0	0	0	0	0	0
	5.0 cm	0	0	0	0	0	0	0	0	0	0	0	0	0	0	0	0
	10.0 cm	0	0	0	0.2	0	0	0.1	0.7	0	0	0.2	0.8	0	0	0.4	0.9
	15.0 cm	0	0	0.1	0.3	0	0	0.4	0.8	0	0	0.5	0.9	0	0.2	0.5	1.0
3.0 cm (GTV 4)	1.0 cm	0	0	0	0	0	0	0	0	0	0	0	0	0	0	0	0
	3.0 cm	0	0	0	0	0	0	0	0	0	0	0	0	0	0	0	0
	5.0 cm	0	0	0	0	0	0	0	0	0	0	0	0	0	0	0	0
	10.0 cm	0	0	0	0	0	0	0	0	0	0	0	0	0	0	0	0
	15.0 cm	0	0	0	0	0	0	0	0.2	0	0	0	0.2	0	0	0	0.2

### 3.D Maximum distance to secure clinical PTV margin for different tolerance values

The maximum distance from the isocenter, at which the 0.1‐cm PTV margin is sufficient to meet each dose reduction tolerance value for different GTV diameters, was shorter with decreasing diameter and increasing 6DoF setup error (Table 6).

**Table 6:** The maximum distance [cm] of the clinical PTV margin that secured each tolerance value of dose coverage with setup error.

Tolerance value	GTV dia.	Translational error															
		0 mm				0.3 mm				0.5 mm				1.0 mm			
		Rotational error															
		0.5°	1.0°	1.5°	2.0°	0.5°	1.0°	1.5°	2.0°	0.5°	1.0°	1.5°	2.0°	0.5°	1.0°	1.5°	2.0°
3%	1.0 cm (GTV 1)	5.5	3.3	2.4	1.8	0	0	0	0	0	0	0	0	0	0	0	0
	1.5 cm (GTV 2)	7.5	4.3	3.2	2.5	3.6	2.3	1.8	1.5	0	0	0	0	0	0	0	0
	2.0 cm (GTV 3)	11.0	5.4	4.0	3.2	6.9	4.0	3.0	2.5	0	0	0	0	0	0	0	0
	3.0 cm (GTV 4)	19.2	8.9	6.6	5.1	14.4	6.8	4.9	3.9	9.4	5.2	3.8	3.2	0	0	0	0
5%	1.0 cm (GTV 1)	7.6	3.8	2.5	1.9	5.7	3.3	2.4	1.8	0	0	0	0	0	0	0	0
	1.5 cm (GTV 2)	10.9	5.5	3.6	2.7	10.4	5.4	3.4	2.5	5.5	3.2	2.5	2.1	0	0	0	0
	2.0 cm (GTV 3)	14.3	7.1	4.8	3.6	14.0	6.9	4.6	3.4	11.9	6.0	4.3	3.2	0	0	0	0
	3.0 cm (GTV 4)	21.4	10.7	7.1	5.4	21.2	10.5	6.9	5.2	17.5	9.6	6.2	4.5	10.6	5.7	4.1	3.3
10%	1.0 cm (GTV 1)	11.9	7.6	5.1	3.8	9.8	7.2	4.7	3.2	9.3	6.7	4.3	2.9	0	0	0	0
	1.5 cm (GTV 2)	22.0	11.0	7.4	5.5	20.2	9.8	6.7	4.5	19.4	9.2	6.1	3.9	11.2	5.8	4.3	3.3
	2.0 cm (GTV 3)	24.7	14.4	9.7	7.2	23.2	13.2	8.7	6.2	22.8	12.7	8.2	5.5	21.3	11.2	7.3	5.1
	3.0 cm (GTV 4)	28.2	17.8	15.0	12.9	27.2	16.7	13.8	11.5	26.0	16.2	13.1	10.8	24.8	15.1	11.9	10.2

## 4 DISCUSSION

We evaluated the effects of the 6DoF setup error during SIVMAT on the dose coverage of a GTV while varying both the distance from the isocenter and the diameter of the target with various dose coverage reduction tolerance values. Using a clinically acceptable margin, the 1, 1.5, and 2 cm GTVs cannot meet the 3% tolerance value under any tested conditions, when the translation error equals or exceeds 0.5 mm (Table 6). In addition, the 1, 1.5, and 2 cm GTVs cannot meet the 5% tolerance value under any tested conditions when the translation error equals or exceeds 1.0 mm. For the same margin, when the tolerance value was 10%, the 1.5–3 cm GTVs could meet the tolerance value of dose coverage reduction in most cases, even at distances >15 cm from isocenter, with up to 0.5° rotational error and up to 0.5 mm translation error. Therefore, when the single‐isocenter technique is used for multiple brain metastases, the relationship between the 6DoF setup error, GTV size, and the distance from the isocenter should be considered when setting margins and expectations for potential dose coverage reduction.

Based on clinical data, Roper et al.^21^ observed that a 0.5° rotational error had no significant impact on dose coverage, whereas a 2.0° rotational error had significant effects on the dose to 95% of the PTV (D_95%_) and the volume covered by 95% of the prescribed dose (V_95%_) of the PTV using a single‐isocenter technique. Their qualitative finding that the reduction of dose coverage increases when the distance between the isocenter and target increases and the target diameter reduces is consistent with our present findings. The novelty of our study is the quantitative evaluation of the important parameters (i.e., the GTV diameter and the distance from the isocenter) in addition to the 6DoF setup error. Our results demonstrated that the minimal coverage‐based margin needed to maintain GTV coverage varied for different coverage reduction tolerance values. The coverage‐based margin was obtained by changing the tolerance values to 3%, 5%, and 10% dose coverage reduction. When setup error is introduced, margins must increase to achieve fixed coverage as targets get smaller or are located further from the isocenter (Tables 3, 4, and 5). In order to choose a coverage‐based margin, it is necessary to know what tolerance for dose coverage reduction is acceptable. However, the tolerance value for reduction in dose coverage needs to be determined based on clinical data obtained from SIVMAT. Further research concerning the tolerance value is needed to determine how the reduction of dose coverage affects the target control rate and the side effects on normal tissue.

In SRS for brain metastases, the clinical PTV margin has frequently been set by adding 0.1 cm to the GTV diameter to concentrate the high dose on the GTV and minimize the doses to surrounding normal tissue.^8^, ^23^, ^24^, ^25^, ^26^ Herein, the coverage‐based margin greater than 0.1 cm is necessary in many of the conditions in this study. In brain SRS on multiple isocenters, a high dose to the brain could cause necrosis by extending the PTV margin.^23^, ^24^, ^25^, ^26^, ^27^ It has also been reported that a PTV margin exceeding 0.1 cm does not affect the local control rate but can have side effects such as radiation necrosis in the brain after SRS.^28^, ^29^ Other researchers have reported that a 0.1 cm PTV was appropriate in SRS.^21^, ^30^ Thus, for patients with brain metastases, it would be advisable to apply SIVMAT so that a 0.1‐cm PTV margin ensures the dose coverage within the tolerance value at a given distance from the isocenter to the target. We observed that the maximum distance at which the clinical PTV margin satisfied each tolerance value for each GTV diameter decreased with a decreasing diameter and an increasing 6DoF setup error (Table 6).

Imaging guidance systems such as the ExacTrac (BrainLAB, Feldkirchen, Germany) and SyncTraX FX4 (Shimadzu, Kyoto, Japan) and cone beam computed tomography, are used for cranial SRS in clinical settings, as they improve the accuracy of the patient localization setup.^18^, ^31^, ^32^, ^33^, ^34^, ^35^ It was reported that these systems can correct the 6DoF setup errors with accuracy within approximately 0.5° and 0.5 mm.^34^, ^35^, ^36^ Therefore, if patient setup corrections < 0.5° and 0.5 mm are not possible due to device‐specific uncertainty, it would be prudent not to use SIVMAT when the GTV diameter is as small as 1.5 cm. When the likely patient localization set up error is no more than 0.5° and 0.5 mm, the maximum distances at which the clinical PTV margin satisfied the 5% tolerance values were 5.5 cm for 1.5 cm diameter, 11.9 cm for 2 cm diameter and 17.5 cm for 3 cm diameter GTV. The GTV dose coverage may be reduced by 5% or more for targets that are <1.5 cm in diameter and >5.5 cm from the isocenter, even with small 6DoF setup errors (0.5° and 0.5 mm) with SIVMAT. This study has two limitations. First, the dose calculation differs as path lengths, electron densities, and the penumbra of dose distribution change, and immobilization systems move. Our present calculations were purely geometric. The effect of the 6DoF setup error would be evaluated more accurately by considering them. Second, we also evaluated the effect of 6DoF setup error on the target dose coverage in SIVMAT by using a simulated spherical target, but the shape of a target in clinical settings is not necessarily a sphere.^37^, ^38^ It is thought that the dose coverage may be further reduced by simulating the shape of the target instead of assuming it to be a sphere. The shape of the target must be considered in future evaluations of the effect of the 6DoF setup error in SIVMAT.

## 5 CONCLUSION

In this study, we evaluated the effect of 6DoF error in the patient setup on the dose distribution in SIVMAT. We found that the increasing setup error on SRS for multiple brain metastases with a single‐isocenter technique increases the risk of the dose missing the tumor with an increase in the distance from the isocenter and a decrease in the tumor size.

## 6 AUTHOR CONTRIBUTION STATEMENT

H. Nakano and S. Tanabe designed the study. H. Nakano, S. Utsunomiya, T. Yamada, and R. Sasamoto performed the experiments. T. Nakano, H. Saito, T. Takizawa, and H. Sakai advised on the content of the study. A. Ohta, E. Abe, M. Kaidu, and H. Aoyama supervised and reviewed the manuscript.

## CONFLICT OF INTEREST

The authors have no conflict of interest to declare.
